# Desensitization, Trafficking, and Resensitization of the Pituitary Thyrotropin-Releasing Hormone Receptor

**DOI:** 10.3389/fnins.2012.00180

**Published:** 2012-12-13

**Authors:** Patricia M. Hinkle, Austin U. Gehret, Brian W. Jones

**Affiliations:** ^1^Department of Pharmacology and Physiology, University of Rochester Medical CenterRochester, NY, USA; ^2^Department of Science and Mathematics, National Technical Institute for the Deaf, Rochester Institute of TechnologyRochester, NY, USA; ^3^Department of Pharmacology, University of WashingtonSeattle, WA, USA

**Keywords:** dephosphorylation, desensitization, internalization, phosphorylation, Rab, thyrotropin-releasing hormone, trafficking, TRH receptor

## Abstract

The pituitary receptor for thyrotropin-releasing hormone (TRH) is a calcium-mobilizing G protein-coupled receptor (GPCR) that signals through Gq/11, elevating calcium, and activating protein kinase C. TRH receptor signaling is quickly desensitized as a consequence of receptor phosphorylation, arrestin binding, and internalization. Following activation, TRH receptors are phosphorylated at multiple Ser/Thr residues in the cytoplasmic tail. Phosphorylation catalyzed by GPCR kinase 2 (GRK2) takes place rapidly, reaching a maximum within seconds. Arrestins bind to two phosphorylated regions, but only arrestin bound to the proximal region causes desensitization and internalization. Phosphorylation at Thr365 is critical for these responses. TRH receptors internalize in clathrin-coated vesicles with bound arrestin. Following endocytosis, vesicles containing phosphorylated TRH receptors soon merge with rab5-positive vesicles. Over approximately 20 min these form larger endosomes rich in rab4 and rab5, early sorting endosomes. After TRH is removed from the medium, dephosphorylated receptors start to accumulate in rab4-positive, rab5-negative recycling endosomes. The mechanisms responsible for sorting dephosphorylated receptors to recycling endosomes are unknown. TRH receptors from internal pools help repopulate the plasma membrane. Dephosphorylation of TRH receptors begins when TRH is removed from the medium regardless of receptor localization, although dephosphorylation is fastest when the receptor is on the plasma membrane. Protein phosphatase 1 is involved in dephosphorylation but the details of how the enzyme is targeted to the receptor remain obscure. It is likely that future studies will identify biased ligands for the TRH receptor, novel arrestin-dependent signaling pathways, mechanisms responsible for targeting kinases and phosphatases to the receptor, and principles governing receptor trafficking.

## Introduction

**Figure 1:**
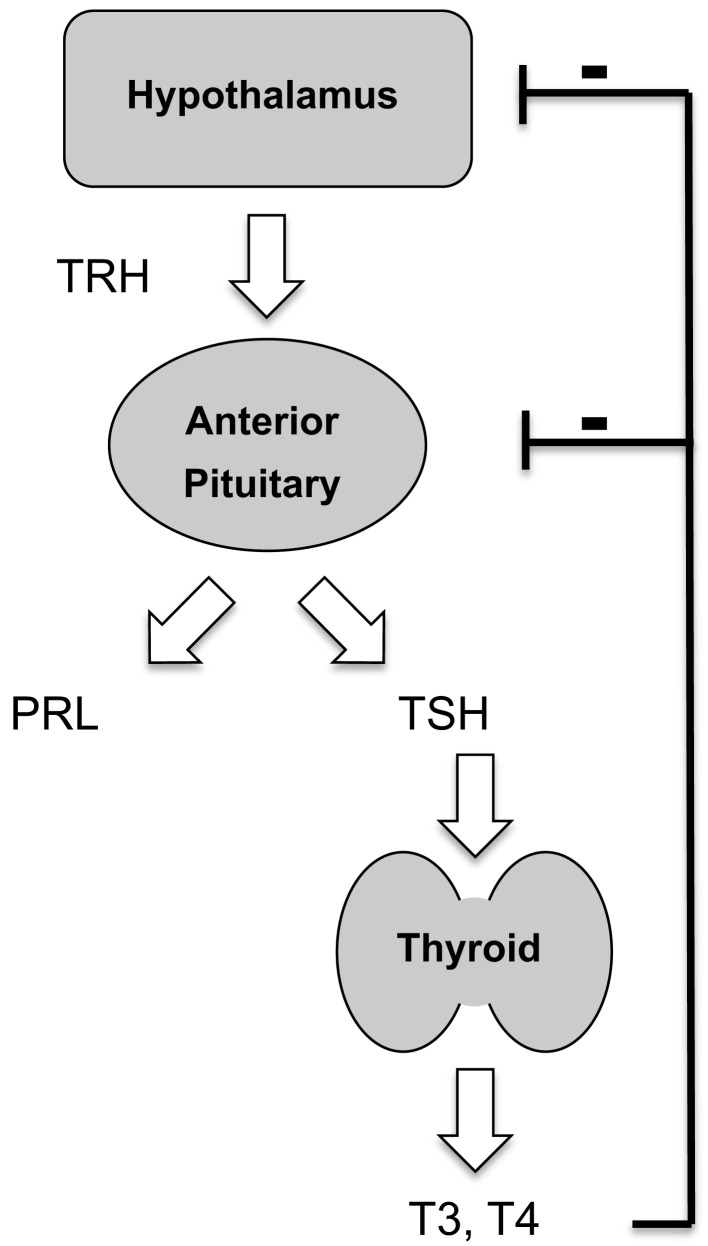
**Endocrine role of thyrotropin-releasing hormone**. TRH, acting via the type 1 TRH receptor, controls secretion from pituitary thyrotrophs and lactotrophs. Thyroid hormones exert negative feedback control.

Thyrotropin-releasing hormone (TRH), a hypothalamic tripeptide, sits atop the hypothalamic/pituitary/thyroid axis, stimulating release of thyrotropin from the anterior pituitary gland. TRH also stimulates prolactin secretion (Figure 1). Initially characterized in clonal rat pituitary cell lines, TRH binding sites were later identified in brain membranes (Hinkle and Tashjian, 1973; Burt and Snyder, 1975). The link between TRH receptors and G proteins was made at a time when heterotrimeric G proteins were thought to transduce signals only from receptors coupled to adenylyl cyclase (Hinkle and Kinsella, 1984; Hinkle and Phillips, 1984). It quickly became clear that TRH receptors belong to the G protein-coupled receptor (GPCR) superfamily. A single TRH receptor gene has been found in humans and higher mammals and two genes encoding homologous receptors, TRHR1 and TRHR2, in rodents (Sun et al., 2003). TRHR1 predominates in the anterior pituitary gland while both TRHR1 and TRHR2 are found in rodent CNS (O’Dowd et al., 2000). Thyroid hormones exert powerful feedback inhibition over the TRH response system by inhibiting TRH synthesis and processing in TRH neurons in the paraventricular region of the hypothalamus and decreasing TRH receptors and responses in the pituitary gland (Gershengorn, 1978; Perrone and Hinkle, 1978; Hinkle and Goh, 1982; Segerson et al., 1987; Fekete and Lechan, 2007; Costa and Hollenberg, 2012). Prolonged hypothyroidism leads to a 40-fold increase in TRH receptor mRNA levels in pituitary glands (Costa et al., 2012). In addition, pyroglutamyl peptidase, a highly specific TRH-degrading ectoenzyme, is dramatically increased in hyperthyroid animals (Schomburg and Bauer, 1997; Marsili et al., 2011).

Genetic deletion of either the TRH peptide precursor or TRHR1 results in central hypothyroidism and mild growth retardation in mice, consistent with the established role of TRH in thyroid physiology (Yamada et al., 1997; Rabeler et al., 2004; Zeng et al., 2007). Interestingly, loss of the TRH receptor appears to result in a more severe phenotype than loss of the TRH peptide, possibly because the TRH receptor displays physiologically significant constitutive activity. Mice lacking TRHR1 also exhibit low prolactin and hyperglycemia, as well as increased anxiety and depression. Some of these behavioral effects may be secondary to hypothyroidism. Mice lacking TRHR2 display a subtle phenotype consistent with depression and decreased anxiety (Sun et al., 2009). In humans, absence of the TRH receptor causes low free T3 and free T4 with thyroid stimulating hormone (TSH) levels inappropriately low for the degree of hypothyroidism (Bonomi et al., 2009). Lack of a TRH receptor does not result in infertility or failure to lactate.

This review focuses on aspects of TRH receptor signaling that have been elucidated in either pituitary cell models expressing endogenous receptors (pituitary tissue, primary cultures of pituitary cells, cell lines derived from pituitary tumors) or generic cell lines expressing transfected TRH receptors (HEK293, CHO, COS, Hela). In rat anterior pituitary tissue, TRH receptor mRNA is found not only in TSH-secreting cells but also in prolactin- and/or growth hormone-secreting cells (Konaka et al., 1997). Although the great majority of pituitary cells that bind rhodamine-labeled TRH and respond to TRH with an increase in intracellular calcium stain for prolactin or TSH-β, responses are occasionally observed in unexpected cell types (Ashworth et al., 1995; Villalobos et al., 1997; Yu et al., 1998). Signal transduction of endogenous TRH receptors has not been characterized in neurons, where no suitable model system has been identified. Even though there is only one TRH receptor in higher mammals, that receptor may be regulated quite differently in pituitary cells and neurons because of different patterns of TRH stimulation and different sets of G protein subunits, effectors, receptors kinases, and phosphatases, arrestins, and downstream targets.

**Figure 2:**
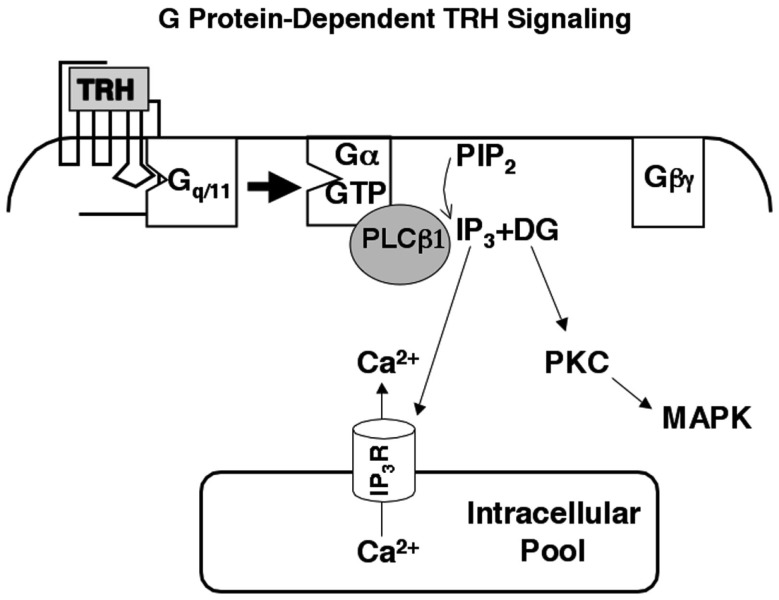
**Thyrotropin-releasing hormone signaling**. TRH mobilizes intracellular calcium and activates protein kinase C through a well-characterized, Gq/11-dependent pathway. Phospholipase Cβ1, which is activated primarily by Gα-GTP subunits, is the major effector in pituitary cells. Intracellular calcium and protein kinase C initiate multiple responses including activation of MAP kinase and regulation of ion channels.

As outlined in Figure 2, TRH receptors are textbook calcium-mobilizing receptors: they are coupled to Gq and G11, which activate phospholipase Cβ (PLCβ), hydrolysis of phosphatidylinositol (4,5)bisphosphate, and release of the second messengers inositol (1,4,5)triphosphate (IP3) and diacylglycerol (DG) that in turn mobilize intracellular calcium and activate protein kinase C (Drummond et al., 1984; Drummond, 1985; Gershengorn, 1989). Two of the four forms of phospholipase Cβ (PLCβs 1 and 3) are found in anterior pituitary tissue, and calcium responses to TRH are intact in mice lacking PLCβ3 (Romoser et al., 2001). Thus PLCβ1, which is stimulated by Gα_q/11_-GTP but not Gβγ, generates second messengers in response to TRH. Maximal concentrations of intracellular free calcium are attained at concentrations of TRH at least an order of magnitude below the apparent *K*_d_ of the receptor and the EC_50_ for IP3 production. These initial events are followed by complex changes that result in depolarization and a sustained influx of extracellular calcium through voltage-gated L-type calcium channels (Hinkle et al., 1996; Barros et al., 1997). When TRH receptors are expressed in embryonic fibroblasts from mice lacking Gα_q_ and Gα_11_, TRH does not stimulate any increase in intracellular calcium.

The TRH receptor is a rhodopsin family GPCR with typical features including seven transmembrane domains, several extracellular glycosylation sites, an essential disulfide bond between the first and second extracellular loops, a fairly short third intracellular loop, and a cytoplasmic tail. The receptor has a canonical (D/E)RY at the cytoplasmic end of the third transmembrane segment, the usual NPxxY at the cytoplasmic end of the seventh transmembrane helix, and an intracellular eighth helix anchored by two palmitoylated Cys residues in the carboxyl tail (Du et al., 2005). Except for the most distal region, the cytoplasmic tail of the receptor is conserved among species. The C-terminal amino acids of numerous GPCRs form classical PDZ ligands, sequences that can interact with proteins bearing a PDZ domain; PDZ is an acronym for three proteins containing the domain, PSD98, Dlg1, and zo-1 (Romero et al., 2011). TRH receptors, however, do not contain sequences predicted to interact with PDZ domains. Using a combination of experimental data obtained with receptor mutants, multiple ligands, and molecular modeling, Gershengorn and his colleagues have developed a sequential model for receptor binding and activation in which the TRH tripeptide first interacts weakly with extracellular loops and then moves deeper into the receptor and contacts transmembrane domains to form a high affinity complex. The conformational changes that occur upon receptor activation involve movements in transmembrane helices 5 and 6 and the third intracellular loop that result in G protein coupling (Gershengorn and Osman, 1996).

## Desensitization of the TRH Receptor

**Figure 3:**
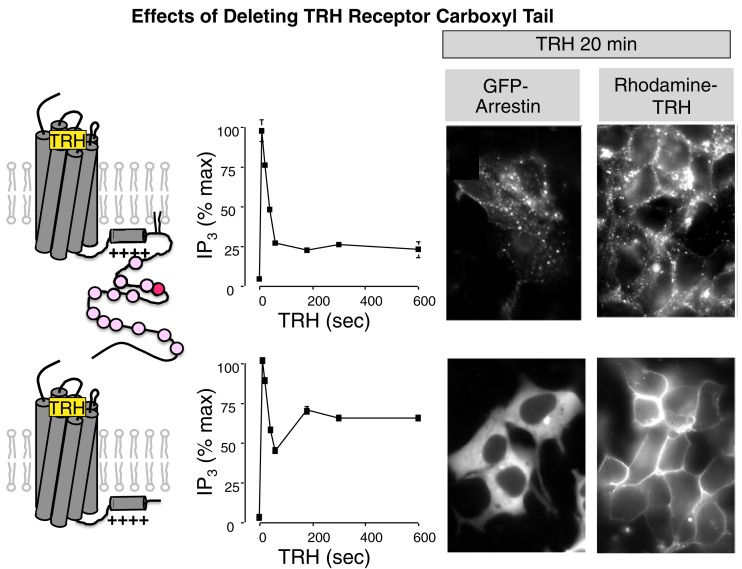
**Importance of the TRH receptor carboxyl terminus**. Wildtype TRH receptors or receptors deleted at amino acid 334 were incubated with TRH and IP3 mass was measured at intervals (graphs). To visualize arrestin, receptors were expressed with GFP-labeled arrestin3. To visualize receptors, cells were incubated with rhodamine-labeled TRH. GFP-arrestin is recruited to full-length TRH receptors and co-internalizes with them but it does not interact with truncated receptors, which do not internalize.

Thyroid stimulating hormone displays a circadian pattern that is attributed to rhythmic release of hypothalamic TRH. As a consequence, desensitization and resensitization of the TRH signaling pathway are likely to play central roles in regulating TRH actions *in vivo*. TRH rapidly stimulates a large increase in IP3 mass, providing a proximal, easily quantified response. Measurements of IP3 production have established that the TRH receptor undergoes classical desensitization. Continuous application of TRH leads to a transient burst of IP3 that peaks within seconds and falls within a minute, though to levels that remain above baseline (Drummond et al., 1984; Gershengorn and Osman, 1996; Yu and Hinkle, 1997, 1998). When cells are exposed to TRH intermittently, the size of the IP3 response diminishes with successive stimulations. The extent of desensitization depends on the method used to measure it and the cell type under study (Falck-Pedersen et al., 1994). Decoupling the receptor from G proteins contributes to the transient nature of the IP3 elevation, which is more sustained in cells expressing a TRH receptor that lacks most of the cytoplasmic tail and is thereby spared from usual desensitization processes (Yu and Hinkle, 1998; Jones and Hinkle, 2005), as shown in Figure 3. Changes further downstream in the signal pathway can also decrease TRH responses by mechanisms including protein kinase C-mediated inhibition of phospholipase Cβ and slow refilling of intracellular calcium stores (Yu and Hinkle, 1997). TRH receptors were the first GPCRs shown to undergo what is now termed homologous downregulation (Hinkle and Tashjian, 1975; Gershengorn, 1978). Incubation with TRH decreases the number of TRH binding sites without changing receptor affinity in pituitary GH3 cells. The molecular basis for downregulation is still not completely understood.

Like many rhodopsin family GPCRs (Bulenger et al., 2005; Milligan, 2009; Lohse, 2010), TRH receptors form oligomers when they are overexpressed in non-native cells (Kroeger et al., 2001; Hanyaloglu et al., 2002; Zhu et al., 2002). Bioluminescence resonance energy transfer (BRET), co-precipitation and biochemical analyses have all been used to document TRH receptor homodimers, and the two rodent TRH receptors (TRHR1 and TRHR2) form heterodimers (Hanyaloglu et al., 2002). TRH increases the apparent fraction of receptors in dimers quantified by all of these techniques, perhaps because TRH causes receptors to concentrate in clathrin-coated pits and endosomes. Truncated receptors that do not recruit arrestin or internalize can form dimers constitutively but not in response to TRH (Zhu et al., 2002). When receptors are coerced to form oligomers by addition of a chemical dimerizing agent, signaling is not altered but internalization is accelerated and recycling blunted (Song and Hinkle, 2005). It has been difficult to assess the physiological relevance of TRH receptor dimers because no mutations that prevent oligomerization have been identified and available methods are inadequate to characterize endogenous receptors in pituitary cells. Recent studies (Whorton et al., 2007) have provided compelling evidence that at least some rhodopsin family GPCRs can activate G proteins as monomers, yet there are clear examples of receptors that form heterodimers with unique pharmacology (Jordan and Devi, 1999). One possibility is that that only one member of a GPCR pair signals to a single G protein. The TRHR1/TRHR2 heterodimer interacts with arrestins and internalizes in a different pattern from either receptor alone (Pfleger et al., 2004), but the intriguing possibility that the TRH receptor forms heteromers with other GPCRs to impart novel pharmacology has not been explored.

## Overview of GPCR Receptor Desensitization

Most GPCRs become phosphorylated after they are activated, and the TRH receptor is no exception. GPCR phosphorylation is catalyzed by members of a family of Ser/Thr protein kinases, GPCR kinases, or GRKs. There are seven GRKs, four of them widely expressed (GRKs 1, 2, 5, and 6; Moore et al., 2007; Gurevich et al., 2012). What sets the GRKs apart from other kinases is their ability to discriminate between GPCRs in inactive versus activated conformations. They act preferentially on agonist-occupied and constitutively active receptors and do not have strict requirements for the amino acid sequence surrounding phosphorylation sites. There are important differences in how different GRKs are recruited to the plasma membrane, however, and examples of GPCRs that are phosphorylated at different sites by different GRKs. GRKs exert a number of kinase-independent effects on GPCR signaling and are known to phosphorylate substrates other than receptors (Moore et al., 2007; Premont and Gainetdinov, 2007; Evron et al., 2012; Gurevich et al., 2012). GPCRs can also be phosphorylated by kinases that become activated in response to signaling such as cAMP-dependent protein kinase and protein kinase C. Phosphorylation carried out by such downstream kinases can influence GPCR-G protein coupling, desensitization, and trafficking.

Once activated and phosphorylated, most GPCRs bind to arrestin, which interrupts the interaction between an activated receptor and its cognate G protein and terminates signaling via the G protein-mediated pathway. Arrestins 2 and 3 (also referred to as β-arrestin1 and β-arrestin2) are ubiquitously expressed. Like G proteins and GRKs, arrestins distinguish between inactive and activated receptor conformations. Arrestins bind preferentially to activated GPCRs; they also contain a positively charged pocket that interacts strongly with negatively charged phosphates (Gurevich and Gurevich, 2006; DeWire et al., 2007; Moore et al., 2007; Premont and Gainetdinov, 2007; DeFea, 2011; Shenoy and Lefkowitz, 2011). The arrestins therefore tend to bind with highest affinity to receptors that are both activated and phosphorylated. When arrestin engages receptor, a buried helical region in the arrestin carboxyl terminus undergoes a major translocation to expose binding sites for AP2 and clathrin, targeting the arrestin-receptor complex to coated pit regions of the membrane destined to pinch off into endosomes in a dynamin-dependent process (Moore et al., 2007; Shenoy and Lefkowitz, 2011).

Arrestin is a scaffolding protein capable of anchoring a wide array of kinases, phosphatases, and other proteins to the arrestin-GPCR complex (Xiao et al., 2007; DeFea, 2011). A rapidly expanding body of literature shows that arrestin-GPCR complexes can initiate alternate signaling pathways, notably those leading to MAP kinase activation (Lefkowitz and Shenoy, 2005; Luttrell and Gesty-Palmer, 2010; Reiter et al., 2012). The Lefkowitz group has proposed that the phosphorylation pattern of a GPCR can act as a barcode in which different phosphosites recruit different arrestins to turn on different signal pathways (Liggett, 2011; Nobles et al., 2011). This model is appealing because it adds tremendous diversity to GPCR signaling, and it is supported by evidence that arrestin2 and arrestin3 bind preferentially to distinct phophosites on several receptors (Ren et al., 2005). There are also many examples of what are termed biased ligands that favor particular pathways (Rajagopal et al., 2010; Reiter et al., 2012). For example, biased ligands may preferentially activate G protein or arrestin cascades, activate one arrestin signaling function but not another (such as kinase activation and internalization), or act as an agonist in one pathway and an antagonist in another. Biased agonists and antagonists open new doors for the development of therapeutically useful GPCR ligands.

G protein-coupled receptors have been broadly divided into two groups based on their interactions with arrestins (Oakley et al., 1999, 2000). Class A receptors bind arrestin 3 more strongly than arrestin 2 or visual arrestin, internalize without associated arrestin, and recycle rapidly. Examples include the well-characterized β2-adrenergic receptor, the μ-opioid receptor and dopamine D1 receptor. Class B receptors bind well to both arrestin 2 and arrestin 3, internalize with arrestin, and traffic to lysosomes where they undergo intracellular degradation or slow recycling. Examples include the V2 vasopressin receptor, type 1a angiotensin II receptor, and the TRH receptor.

## Phosphorylation of TRH Receptors

**Figure 4:**
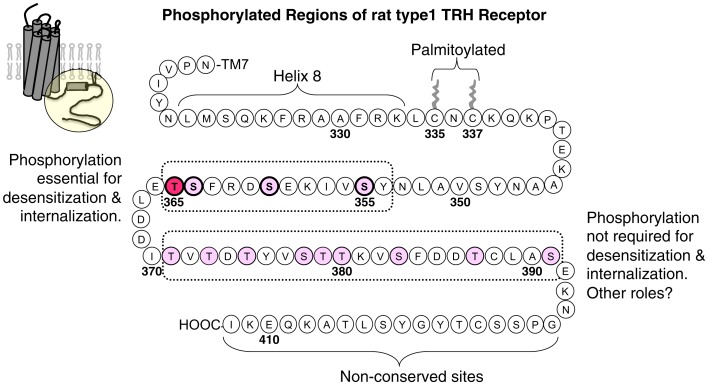
**Thyrotropin-releasing hormone receptor phosphorylation sites**. The circle outlines the portion of the rat TRH receptor depicted. Ser/Thr residues are shown in pink or red in regions known to undergo TRH-dependent phosphorylation. Phosphorylation at Thr365, in red, is essential for desensitization and internalization, and phosphorylation at other sites between amino acids 355 and 365 has been documented and shown to contribute to these processes. TRH also stimulates phosphorylation at sites from 371 to 391, although the exact phosphosites are not known. Phosphorylation in this region is not essential for desensitization and internalization, but it may be important for different, as yet unidentified signaling functions. Phosphorylation in the non-conserved distal region has not been examined.

Thyrotropin-releasing hormone receptors become phosphorylated rapidly once they are activated. Receptor phosphorylation has been demonstrated by ^32^P incorporation, an upward mobility shift on gel electrophoresis, and reactivity with phosphosite-specific antibodies (Hanyaloglu et al., 2001; Zhu et al., 2002; Jones and Hinkle, 2005; Jones et al., 2007). All of these changes are reversed by phosphatase treatment. Phosphorylation sites in the TRH receptor have been partially mapped by characterizing ^32^P incorporation and antibody reactivity of wildtype receptors and receptors lacking various Ser and Thr residues in the cytoplasmic tail. Jones et al. generated antisera against multiply phosphorylated peptides from five different conserved regions of the TRH receptor tail and validated their specificity (Jones and Hinkle, 2008). None of the antibodies recognize TRH receptors from unstimulated cells, but four of them react with receptors from TRH-treated cells. Endogenous TRH receptors in pituitary cells are phosphorylated strongly at residues between amino acids 355 and 365 and less efficiently at two regions farther downstream. Current information about phosphosites in the TRH receptor C-terminus is presented in Figure 4. As discussed below, phosphorylation of Thr365 is particularly important for arrestin recruitment, internalization, and desensitization. There is no evidence for phosphorylation at Tyr residues. The distal regions of the TRH receptor are more heavily phosphorylated when the receptor is expressed in HEK293 or CHO cells compared to native receptors. The finding of different site usage in different settings suggests a need for caution when charactering phosphorylation of overexpressed epitope-tagged GPCRs in heterologous cells, an approach that is normally required for analysis by mass spectrometry.

Thyrotropin-releasing hormone receptor phosphorylation does not appear to be hierarchical because the rates of phosphorylation are the same for different phosphosites. TRH does not promote ^32^P incorporation into receptors truncated before the palmitoylation site and TRH does not alter mobility of truncated receptors, leading to the conclusion that either intracellular loops are not phosphorylated in response to TRH or phosphorylation in the intracellular loops somehow requires the receptor tail. Following TRH addition, nearly all receptors appear to be phosphorylated based on the mobility shift and near quantitative immunoprecipitation with phosphosite-specific antibodies (Jones et al., 2007).

**Figure 5:**
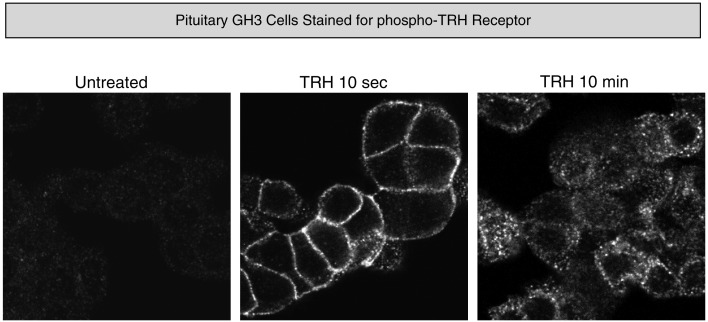
**Thyrotropin-releasing hormone-dependent phosphorylation of endogenous TRH receptors**. Rat pituitary GH3 cells were stained with affinity-purified antibody specifically recognizing phosphosites at amino acids 355–365 of the TRH receptor before and after TRH exposure.

In pituitary cells, endogenous receptors on the plasma membrane are not detectably phosphorylated in the basal state, but they become strongly phosphorylated within 10 s of TRH addition and endocytosis of phospho-receptors is readily apparent by 10 min (Figure 5). Interestingly, when rat pituitary tissue from an untreated animal is examined, phosphorylated TRH receptor is clearly visible. The intensity of the phospho-receptor signal increases greatly minutes after animals are injected with TRH and, as expected, the signal is seen cells that stain for TSH-β and prolactin (Jones et al., 2007).

Thyrotropin-releasing hormone receptor phosphorylation is exceedingly fast, with a half-time of 0.2 min (Jones et al., 2007; Gehret and Hinkle, 2010). This rapid phosphorylation is unusual among GPCRs and marks the TRH receptor as a superior substrate. Rapid phosphorylation is not simply a consequence of the sequence of the receptor tail. The transmembrane helices and intracellular loops of the TRH receptor are likewise important for rapid phosphorylation of the receptor tail even though they are not phosphorylated themselves. A chimeric TRH receptor with the β2-adrenergic receptor tail is phosphorylated at β2-adrenergic receptor GRK sites with kinetics typical of the TRH receptor, not the β2-adrenergic receptor (Gehret and Hinkle, 2010). The rate-limiting step for TRH receptor phosphorylation may be recruitment of GRKs to the activated receptor. In support of this idea, dimerization of a mutant TRH receptor that cannot undergo activation with a truncated TRH receptor that signals well but cannot undergo phosphorylation results in phosphorylation of the signaling-incompetent partner (Song et al., 2007). This is most easily explained if the activatable receptor recruits a kinase that acts on its partner in the receptor dimer. The predicted amphipathic eighth helix between the canonical NPxxY at the end of the seventh transmembrane domain and the palmitoylated Cys-X-Cys motif is not phosphorylated but the positively charged residues in this region are essential for phosphorylation at downstream sites and subsequent internalization (Gehret et al., 2010).

Considerable evidence points toward GRK2 as the kinase responsible for TRH receptor phosphorylation. Phosphorylation is inhibited by dominant negative, kinase-dead forms of GRK2 and by siRNAs targeting GRK2 (Jones and Hinkle, 2005; Jones et al., 2007). The effects of siRNA knockdown are seen both in pituitary cells and heterologous cells expressing transfected receptors. In addition, paroxetine, which has recently been recognized as an effective and relatively specific GRK2 inhibitor, delays phosphorylation of the TRH receptor (Thal et al., 2012). In keeping with the rapid rate of TRH receptor phosphorylation, GRK2 translocates to membrane fractions within 10 s of TRH addition (Jones and Hinkle, 2005). All of these experiments implicate GRK2 in TRH receptor phosphorylation, but because GRK2 knockdown and inhibition do not block receptor phosphorylation completely it seems likely that other GRKs are also able to phosphorylate the receptor.

Inhibitor data suggest that casein kinase II, which is predicted to phosphorylate Thr365 and two other downstream sites, may contribute to receptor phosphorylation (Hanyaloglu et al., 2001). Pharmacological activation of conventional protein kinase C isoforms leads to weak phosphorylation of some but not all sites in the TRH receptor tail, but protein kinase C inhibitors do not alter TRH-dependent phosphorylation (Jones et al., 2007). Furthermore, TRH receptor phosphorylation and internalization occur normally in cells lacking Gα_q_ and Gα_11_, indicating that calcium- and diacylglycerol-activated kinases are not essential (Yu and Hinkle, 1999; Jones and Hinkle, 2005). Together these results suggest little role for downstream kinases in TRH receptor phosphorylation.

## Arrestin Interactions with the TRH Receptor

Arrestin interactions with TRH receptors have been monitored by a variety of approaches including translocation of GFP-arrestin (Groarke et al., 1999, 2001; Oakley et al., 1999, 2000; Yu and Hinkle, 1999; Smith et al., 2001; Hanyaloglu et al., 2002), co-precipitation of arrestin and receptor (Jones et al., 2007), effect of arrestin on agonist affinity (Jones and Hinkle, 2005, 2008), and BRET (Kroeger et al., 2001; Hanyaloglu et al., 2002). GFP-arrestin is diffusely localized in the cytoplasm of cells expressing TRH receptors. When TRH is added, GFP-arrestin2 and GFP-arrestin3 move to the plasma membrane within a minute or two (see Figure 3). The ability of TRH receptors to recruit arrestins 2 and 3 places it in the Group B category of GPCRs. Although arrestin translocation is detectable with most GPCRs, arrestin movement seen with the TRH receptor is exceptionally robust (Oakley et al., 2000). GFP-arrestin translocation is not observed in cells expressing TRH receptors lacking palmitoylation sites or truncated before the major phosphorylation sites (Vrecl et al., 1998; Yu and Hinkle, 1998; Hanyaloglu et al., 2001; Smith et al., 2001; Jones and Hinkle, 2005, 2008).

**Figure 6:**
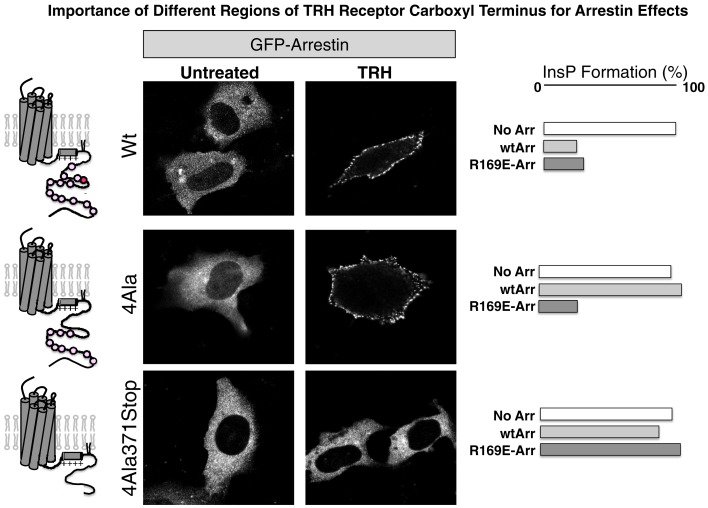
**Arrestin recruitment and arrestin-dependent desensitization**. Cells expressing the receptors shown were incubated with GFP-arrestin and imaged before or after 3 min exposure to TRH. The right panels show TRH-stimulated IP3 production in arrestin-null cells expressing receptor with no arrestin (No Arr), wildtype arrestin 3 (wtArr), or R169E-Arr, which binds activated GPCRs in a phosphorylation-independent fashion. TRH receptors are: Wt, wildtype; 4Ala, Ala substituted for 4 Ser/Thr residues from amino acids 355 through 365; and 4Ala-371Stop, 4Ala receptor truncated at amino acid 370. Even though arrestin binds robustly to receptors lacking phosphosites at amino acids 355–365, arrestin does not desensitize signaling.

**Figure 7:**
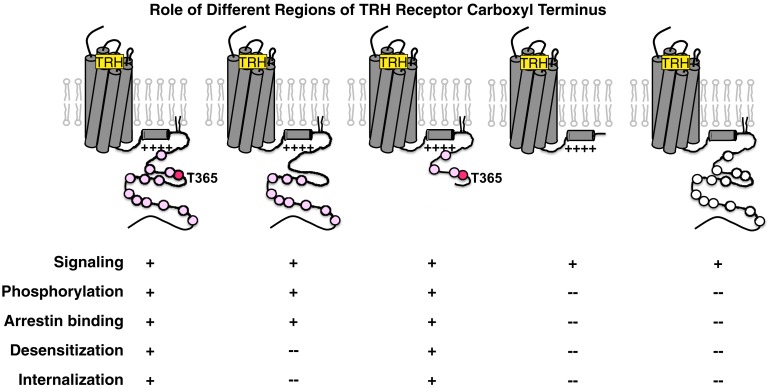
**Importance of different phosphorylated regions of the TRH receptor tail**. Pink circles depict Ser/Thr residues in regions known to be phosphorylated, with the critical Thr365 residue shown in red. From left to right, TRH receptors are: wildtype, 4Ala (Ala-substituted for the 4 Ser/Thr residues from amino acids 355 through 365), 371Stop (receptor truncated at amino acid 370), C335Stop (receptor truncated before palmitoylation sites), and receptor with Gln substitutions for 6 Arg/Lys residues between amino acids 326 and 340. White circles depict Ser/Thr residues that are present but weakly phosphorylated.

As shown in experiments depicted in Figure 6, GFP-arrestin does translocate to receptors with Ala substitutions for the four Ser and Thr residues between 355 and 365, proving that downstream phosphosites are sufficient to bind arrestin (Jones and Hinkle, 2008). This is important because distal phosphosites are not sufficient for desensitization and internalization, as discussed below. Arrestin co-precipitates with activated TRH receptors even if Ser/Thr residues in the 355–365 region are Ala-substituted, again showing that arrestin interacts with at least two regions of the receptor. BRET studies document a close interaction between the type 1 TRH receptor and arrestins 2 and 3 that is lost if the receptor is truncated before the palmitoylation sites (Pfleger et al., 2004). Figure 7 summarizes current understanding about the importance of different TRH receptor phosphorylation sites.

Because arrestins bind preferentially to activated GPCRs, theoretical considerations dictate that arrestin will increase the affinity of a GPCR for agonists, a prediction borne out by experimental evidence (Gurevich et al., 1997). Consistent with this, the apparent affinity of the potent agonist [^3^H]MeTRH, measured at room temperature or above in intact cells, is highly dependent on arrestin levels (Jones and Hinkle, 2005, 2008). In arrestin2/3 knockout cells, cotransfection with wildtype arrestin increases the apparent affinity for [^3^H]MeTRH 14-fold. Arrestin increases the agonist affinity of truncated and Ala-substituted receptors, supporting the concept of multiple arrestin binding sites and raising the possibility of phosphorylation-independent arrestin interactions.

## Role of Arrestin in TRH Receptor Signaling, Desensitization and Internalization

Thyrotropin-releasing hormone receptors expressed in arrestin2/3 knockout cells generate strong IP3 responses to TRH. TRH-stimulated IP3 levels are much lower in cells expressing arrestin2, arrestin3, or both, proving that arrestin is important for desensitization and that either arrestin is sufficient for this function. As expected, arrestins are not required for G protein-dependent signaling. Arrestins do not desensitize IP3 responses if receptors are missing phosphorylation sites between amino acids 355 and 365. Ala substitution of Thr365 severely impairs TRH receptor desensitization and internalization. In contrast, receptors truncated at amino acid 370 are still subject to arrestin-mediated desensitization (Jones et al., 2007; Jones and Hinkle, 2008).

A similar pattern emerges when internalization is examined. Expression of dominant negative arrestin in normal cells or expression of receptors in arrestin-null cells severely restricts the rate and extent of endocytosis (Hanyaloglu et al., 2002; Jones and Hinkle, 2005, 2008). Endogenous arrestin levels control the desensitization and internalization of heterologously expressed TRH receptors; for example, HEK293 and even more so COS-7 cells have lower concentrations of arrestin and correspondingly less desensitization and internalization than pituitary cells (Falck-Pedersen et al., 1994; Vrecl et al., 1998). Arrestin binding to phosphorylated Thr365 and surrounding sites is absolutely required for internalization, but arrestin binding to phosphosites beyond amino acid 370 is not necessary. The TRH receptor is one of several examples where arrestin can bind to a GPCR without leading to internalization (Krasel et al., 2008). Conversely, expression of the R169E arrestin mutant, which binds activated GPCRs in a phosphorylation-independent fashion, restores TRH-dependent internalization to receptors lacking Thr365 and nearby phosphorylation sites (Hanyaloglu et al., 2001; Jones and Hinkle, 2008; see Figure 6). Time- and temperature-dependent conversion of receptor-bound radiolabeled peptide to an acid-resistant state has often been taken as a measure of receptor endocytosis. It is worth noting that this is not valid for receptor-bound [^3^H]MeTRH (Jones and Hinkle, 2008). Acid resistance precedes internalization and occurs at a reduced level with mutant receptors that do not internalize at all and in cells where internalization is blocked. Endocytosis can be documented readily by microscopy or by the TRH-driven loss of surface binding sites for antibodies to an N-terminal epitope tag on the receptor.

**Figure 8:**
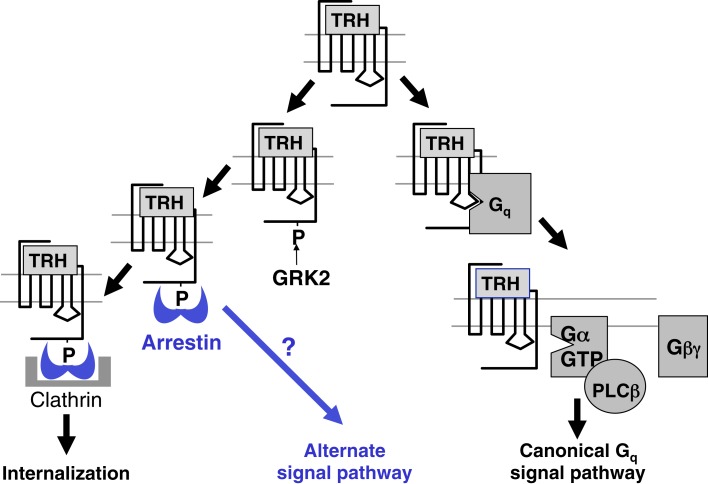
**Arrestin effects on TRH receptor signaling**. Arrestin binds to phosphorylated receptors, terminating receptor-G protein coupling, and initiating receptor endocytosis. Arrestin is a scaffold for other signaling molecules and it is possible that the arrestin-TRH receptor complex controls as yet unidentified pathways.

One question arising from these observations is whether internalization of TRH receptors contributes to early desensitization. To address this question, TRH responses were quantified in settings where receptor endocytosis was effectively blocked: in the presence of hyperosmolar sucrose, in cells infected with vaccinia virus encoding dominant negative dynamin, and in cells expressing receptors truncated just before the palmitoylation site (Yu and Hinkle, 1998). Signaling was also measured in arrestin-null cells transfected with a mutant arrestin that can bind to receptor but is incapable of interacting with clathrin and AP2 (ΔLIELD/F391A-arrestin) and therefore incapable of promoting internalization (Jones and Hinkle, 2005, 2008). Initial Gq/11-mediated responses and subsequent desensitization of intact receptors were unaffected by the lack of internalization, but the lack of a receptor tail resulted in more persistent elevations in IP3 (Yu and Hinkle, 1998). Together these results confirm the importance of arrestin for both desensitization and internalization and prove that internalization is not the cause of desensitization. In highly sensitive assays, persistent IP1 production can be demonstrated for up to an hour after TRH is washed out in HEK293 cells expressing TRH receptors (Boutin et al., 2012). This sustained response does not depend on internalization. The question of whether there are G protein-independent signaling pathways and if so, whether they persist following receptor endocytosis, has not been answered. For example, TRH activates MAP kinase. A strong early activation depends on protein kinase C-dependent raf phosphorylation but not on internalization of the receptor itself, although it does require an intact endocytic machinery (Ohmichi et al., 1994; Smith et al., 2001). Additional work is needed to establish whether the weak sustained activation of MAP kinase generated by TRH depends on arrestin or continues following internalization in pituitary cells. The known and possible additional roles of arrestin are shown schematically in Figure 8.

## Mechanism of TRH Receptor Internalization

Thyrotropin-releasing hormone promotes rapid and extensive internalization of endogenous receptors in pituitary cells (Ashworth et al., 1995). Endocytosis of the activated TRH receptor proceeds via a classical arrestin- and dynamin-dependent pathway and is blocked by pharmacological inhibition of endocytosis and by dominant negative dynamin (Drmota et al., 1998; Yu and Hinkle, 1998). Gq/11-dependent signaling is not required, because internalization occurs normally when TRH receptors are expressed in fibroblasts from mice with genetic deletion of both Gα_q_ and Gα_11_, where there is no calcium response (Yu and Hinkle, 1999). Regions of the cytoplasmic receptor tail important for internalization correspond to regions where phosphorylation takes place (Hanyaloglu et al., 2001, 2002; Jones et al., 2007). Endocytosis is much slower although not entirely absent when the TRH receptor is expressed in cells with either no arrestin or dominant negative arrestins (Vrecl et al., 1998; Groarke et al., 2001; Smith et al., 2001; Hanyaloglu et al., 2002; Jones and Hinkle, 2005). The mechanism of the arrestin-independent component is unknown, but TRH receptors appear to be excluded from caveolae (Rudajev et al., 2005).

Ubiquitination is required for endocytosis of several GPCRs (Hanyaloglu and von Zastrow, 2008; Hislop and von Zastrow, 2011) and a fraction of TRH receptors are ubiquitinated, but ubiquitin is not added to receptors on the plasma membrane and ubiquitination does not occur in response to TRH. Furthermore, receptor endocytosis continues at the non-permissive temperature in cells with a temperature-sensitive E1 ubiquitin-activating enzyme (Cook et al., 2003). It is clear, then, that ubiquitination does not tag TRH receptors for internalization, although ubiquitin-mediated degradation serves an important quality control function during TRH receptor biosynthesis.

## TRH Receptor Trafficking

Trafficking of TRH receptors fused at the C-terminus to GFP has been characterized by several groups (Drmota et al., 1998; Yu and Hinkle, 1999; Scott et al., 2002). Shortly after TRH addition, receptors cluster on the cell surface, and over the course of about 10 min they internalize in pre-assembled vesicles without transferrin receptors or Gα_q_. These earliest vesicles soon merge with others containing transferrin receptors. Some Gα_q_ is removed from the plasma membrane following TRH receptor activation, but this occurs quite slowly and internalized receptors and Gα_q_ are not colocalized (Drmota et al., 1999; Yu and Hinkle, 1999). After 30 min or longer, GFP-labeled TRH receptors are found in much larger vesicles deep inside the cell. TRH receptors internalize together with arrestin (Groarke et al., 1999; Oakley et al., 2000; Smith et al., 2001; Jones and Hinkle, 2008).

As described above, the TRH receptor follows a familiar pattern of phosphorylation, arrestin binding, desensitization, and endocytosis. Much less is understood about what happens next: how is the TRH receptor (and other GPCRs) dephosphorylated, sorted and trafficked back to the plasma membrane or targeted for degradation? In a study that capitalized on the availability of antibodies specific for phosphorylated TRH receptors, trafficking of phosphorylated, and dephosphorylated TRH receptors was monitored during internalization and recycling. Endosomal compartments were identified with GFP-labeled Rab proteins, and movements of fluorescently labeled arrestin were followed simultaneously (Jones and Hinkle, 2009). Cell surface receptors were selectively labeled with antibody against an HA tag on the extracellular N-terminus of the rat TRH receptor (“antibody feeding”) and tracked over time following addition or withdrawal of TRH. Rab5 marks an early endosomal population, and TRH receptors appeared in Rab5-positive vesicles within a few minutes of TRH stimulation. Receptors in these vesicles were almost entirely phosphorylated and associated with arrestin. Over the course of 20 min, receptors moved to vesicles that were both rab4- and rab5-positive, the early sorting endosomes. Both phosphorylated and non-phosphorylated receptors were seen in this pool. Internalized receptors subsequently trafficked to a population of vesicles that were rab4-positive but rab5-negative, typical of rapidly recycling endosomes. These recycling vesicles were enriched in *dephosphorylated* receptors, i.e., receptors that began the experiment on the plasma membrane but were no longer phosphorylated. They were essentially devoid of phosphorylated receptors.

This result raised the question: were receptors able to move into this rab4-positive, rab5-negative “recycling” vesicle population because they were dephosphorylated, or were receptors quickly dephosphorylated once they reached these vesicles? This question was addressed by interrupting normal trafficking with dominant negative rabs. Dominant negative rab5 completely blocked movement of the receptor out of very early endosomes, yet it did not change the rate of receptor dephosphorylation. If dephosphorylation occurred preferentially in a later endosomal population, dominant negative rab5 would have delayed phosphatase action. These results lead to the conclusion that dephosphorylation takes place in sorting endosomes and permits trafficking of the TRH receptor into recycling vesicles.

**Figure 9:**
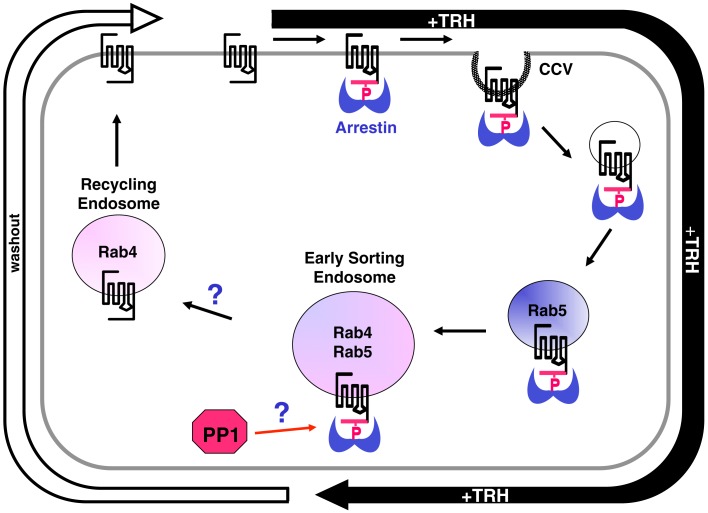
**Intracellular trafficking of TRH receptors**. TRH activation is rapidly followed by receptor phosphorylation, arrestin binding, and recruitment of the arrestin-phospho-receptor complex to clathrin-coated pits that pinch off in a dynamin-dependent process to form clathrin-coated vesicles (CCVs). Vesicles containing phospho-receptor soon merge with rab5-positive vesicles where they colocalize with transferrin receptors. These vesicles gradually merge with others to form larger endosomes rich in rab4 and rab5, early sorting endosomes. After TRH is removed from the medium, but not before, dephosphorylated receptors start to become detectable in rab4-positive but rab5-negative recycling endosomes. Dephosphorylated receptors then recycle to the plasma membrane. Phosphorylated receptor is rarely seen in these fast recycling vesicles. After long incubations with TRH, small amounts of phospho-receptor are detected in rab11 vesicles, considered to be a slow recycling pool. Protein phosphatase 1 (PP1) acts on the TRH receptor, but it is not known how the removal of extracellular TRH triggers receptor dephosphorylation. The mechanism that permits dephosphorylated receptors to move to recycling endosomes, or alternatively the mechanism that prevents phosphorylated receptors from exiting early sorting vesicles, are also not clear.

A small subset of phosphorylated TRH receptors eventually appear in Rab11 vesicles, traditionally viewed as a late recycling compartment, suggesting that the long recycling pathway is taken by some receptors. Rab7 vesicles, which are associated with lysosomes, contained very little TRH receptor. It seems plausible that a fraction of intracellular receptor is degraded with each round of internalization, possibly contributing to the phenomenon of downregulation, but this remains speculative. The trafficking of internalized TRH receptors is summarized in Figure 9.

## Repopulating the Plasma Membrane: Recycling and Recruitment

When cells are incubated with TRH to drive internalization and the hormone is removed, receptors reappear at the plasma membrane with a half-time of 20–30 min based on the amount of radioactive TRH able to bind, the amount of epitope-tagged receptor on the membrane determined by FACS or ELISA, or the amount of GFP-labeled receptor at the surface. These results are consistent with receptor recycling, but the story has recently grown more complicated with the discovery that although the total receptor number at the plasma membrane is restored quite quickly, this is largely due to recruitment of new receptors (Cook and Hinkle, 2004; Jones and Hinkle, 2009). An early piece of evidence appeared when the TRH receptor was fused to a derivative of DS-Red dubbed “Timer.” The unique feature of the Timer protein is that it changes color from red to green with a half-time of 10 h. When TRH was added to drive internalization and then removed to allow recycling, receptors that moved to the plasma membrane were much redder (younger) than those that had been internalized (Cook and Hinkle, 2004). A second approach was an antibody feeding study which showed that intracellular receptors were recruited to the membrane before the internalized receptors had recycled (Jones and Hinkle, 2009). To avoid problems inherent in antibody feeding experiments, TRH receptors were fused at the N-terminus to a 15 amino acid tag that serves as a biotin ligase acceptor. Plasma membrane receptors were selectively labeled with biotin by adding purified bacterial biotin ligase, biotin, and ATP to the medium. TRH receptors on the plasma membrane were essentially the only proteins biotinylated, and biotin-labeled TRH receptor was visualized with fluorescent streptavidin. Again, after TRH-driven endocytosis and several hours of recovery, the surface receptor pool was repopulated initially with recruited rather than recycled (biotin-labeled) receptors (Jones and Hinkle, 2009). Finally, cells were labeled with [^3^H]TRH (not the higher affinity, slower dissociating [^3^H]MeTRH), which internalized with the receptor. When [^3^H]TRH was removed, plasma membrane receptor levels were restored at a time when most [^3^H]TRH still remained inside the cell. In each of these approaches, most internalized receptors eventually returned to the plasma membrane, but only after an hour or more (Jones and Hinkle, 2009). These results provide another question for future study: How are receptors in an intracellular pool prompted to move to the plasma membrane?

## GPCR Dephosphorylation

Once a GPCR is activated, phosphorylated, and bound to arrestin, return to its resting state requires dissociation or degradation of the agonist, dissociation of arrestin and dephosphorylation. With rare exceptions, the phosphatases responsible for GPCR dephosphorylation have not been fully characterized. There are many more GPCRs than Ser/Thr phosphatases, necessitating involvement of enzymes with broad substrate specificity. In mammalian cells, the majority of proteins phosphorylated on Ser and Thr residues are dephosphorylated by members of two ubiquitous enzyme families, protein phosphatase 1 (PP1), and protein phosphatase 2A (PP2A; Barford et al., 1998). These protein phosphatases are multisubunit enzymes that achieve specificity by interacting with a wide array of scaffolding and targeting subunits. The catalytic subunits of PP1s can bind directly to substrates through a number of conserved motifs or localize through targeting subunits. Over 150 proteins have been shown to interact with PP1 (Bollen et al., 2010). PP2As are heterotrimers composed of a catalytic subunit, a scaffolding or structural subunit, and a targeting subunit. On the basis of high sensitivity to inhibitors, the PP2A family of phosphatases has been implicated in the dephosphorylation of numerous GPCRs, and PP1, calcineurin (PP2B), and PP2C for others (Croci et al., 2003; Flajolet et al., 2003; Mao et al., 2005).

Pitcher et al. (1995) reported that the β2-adrenergic receptor was dephosphorylated by an endosomal phosphatase in the PP2A family following internalization. This model predicted an essential role for receptor endocytosis: a GPCR had to cycle through endosomal compartments to be dephosphorylated and resensitized. Subsequent work has uncovered many variants on this theme. Many GPCRs can be dephosphorylated while localized on the cell surface. Dephosphorylation of PKA and GRK sites on the β2-adrenergic receptor can take place on the plasma membrane (Iyer et al., 2006). Some sites on the somatostatin 2A receptor are not dephosphorylated until the receptor has internalized, while others can be dephosphorylated regardless of receptor location; different enzymes appear to be involved (Ghosh and Schonbrunn, 2011). Receptors for several peptide hormones cannot recycle until the peptide is degraded in acidified endosomes in a reaction catalyzed by ectopeptidases that cointernalize with the peptide-receptor complex (Roosterman et al., 2008; Cattaruzza et al., 2009). Although the TRH-degrading enzyme is an ectoenzyme present in pituitary tissue, there is no evidence that it plays a role in TRH receptor cycling.

## TRH Receptor Dephosphorylation

When TRH is removed from the medium or an inverse agonist such as chlordiazepoxide is added, receptor dephosphorylation rapidly ensues (Jones and Hinkle, 2005; Jones et al., 2007; Gehret and Hinkle, 2010, 2012). Once TRH dissociates, receptors return to an inactive conformation, arrestin affinity declines, and arrestin dissociation provides freer access of phosphatases. A number of studies have sought to identify factors controlling the rate of receptor dephosphorylation. The amino acid sequence surrounding the phosphosite does not seem to be a major factor because the rates of TRH receptor dephosphorylation are the same at different phosphosites (Jones et al., 2007). When the β2-adrenergic receptor tail was spliced onto the TRH receptor, the chimeric receptor underwent TRH-dependent phosphorylation at sites that are phosphorylated by GRKs in the β2-adrenergic receptor. The dephosphorylation kinetics of the chimera resembled those of the natural TRH receptor (Gehret and Hinkle, 2010). On the other hand, the location of the TRH receptors is important (Jones et al., 2007; Gehret and Hinkle, 2010). Dephosphorylation occurs more rapidly when receptors are on the plasma membrane than when receptors have undergone endocytosis. In pituitary cells, endogenous receptors are dephosphorylated with a half-time of about 45 s if TRH exposure lasts only a minute (sufficient for complete phosphorylation but not for internalization) but is 3 min if TRH exposure continues for 30 min (when receptors have undergone internalization; Jones et al., 2007). A similar situation holds in heterologous model systems, although dephosphorylation is not nearly as fast.

These data raise an important question: how does removing TRH from the outside of the cell trigger dephosphorylation of receptors in endosomes deep in the cytoplasm? The simplest idea is that some receptors are always at the plasma membrane and as long as TRH is present, they are signaling and somehow maintaining receptor phosphorylation. Removing extracellular TRH terminates signaling and leads to dephosphorylation. If this model is valid, however, the responsible signaling system cannot be traditional Gq/11-mediated activation of PLCβ, because dephosphorylation of internalized receptors is not altered by treatment with an intracellular calcium chelator and protein kinase C inhibitor (Jones et al., 2007).

Recently, the problem of identifying the TRH receptor phosphatase was tackled using an unbiased screen with an siRNA library directed against phosphatase subunits (Gehret and Hinkle, 2012). While this approach had the potential to identify a targeting or scaffolding subunit, only the catalytic subunit of PP1α came up as a bone fide hit in the screen. The results of the screen, which was performed in HEK293 cells, concur with inhibitor effects on dephosphorylation of endogenous TRH receptors in pituitary cells. Dephosphorylation of TRH receptors is powerfully inhibited by calyculin A, which acts on the catalytic subunits of PP1 and PP2A, but insensitive to fostriecin, a highly selective inhibitor of PP2A. Many details, such as the mechanism that targets PP1α to the receptor, remain to be elucidated. The discovery of a specific phosphatase subunit acting on the TRH receptor provides a starting point for dissecting an important aspect of the resensitization process.

## Future Directions

The TRH receptor has been viewed as a prototypical “calcium-mobilizing” GPCR and the mechanisms of TRH signaling have been the subject of intense investigation for decades. More recently, attention has also been focused on events that turn signaling off. In every instance, the pathways involved have turned out to be more complex than anticipated. This review has sought to review recent work and highlight some of the unknowns pertaining to TRH receptor signaling, desensitization, and trafficking. Once activated by hormone, the TRH receptor is phosphorylated in multiple regions; arrestins bind at different phosphosites with different consequences. It seems likely that the TRH receptor signals via its diverse arrestin interactions in ways that are not yet recognized. The trafficking studies described here show that dephosphorylation must take place before internalized TRH receptors can recycle, yet the nature of the interaction between receptor and phosphatase remains elusive. The recent identification of the protein phosphatase involved opens the door for future studies seeking to describe phosphatase targeting mechanisms. The principles governing receptor trafficking are not well understood, and it is unclear how phosphorylated and desphosphorylated receptors are sorted in endosomes. High throughput screening techniques have aided in the discovery of biased ligands with the capacity to control specific aspects of receptor function, and it is to be hoped that designer ligands for TRH receptors will provide new tools for research or even novel drugs for treating thyroid disease. Finally, long awaited information on the structure of the TRH receptor can be expected in the not too distant future.

## Conflict of Interest Statement

The authors declare that the research was conducted in the absence of any commercial or financial relationships that could be construed as a potential conflict of interest.
